# Using machine learning analysis to describe patterns in tissue Doppler and speckle tracking echocardiography in patients with transposition of the great arteries after arterial switch operation^☆^

**DOI:** 10.1016/j.ijcchd.2024.100560

**Published:** 2024-12-20

**Authors:** Covadonga Terol Espinosa de los Monteros, Roel L.F. van der Palen, Jef Van den Eynde, Lukas Rammeloo, Mark G. Hazekamp, Nico A. Blom, Irene M. Kuipers, Arend D.J. ten Harkel

**Affiliations:** a Department of Pediatrics, Division of Pediatric Cardiology, Leiden University Medical Center, Leiden, the Netherlands; b Department of Cardiovascular Sciences, KU Leuven, Leuven, Belgium; c Department of Pediatrics, Division of Pediatric Cardiology, Amsterdam University Medical Center, Amsterdam, the Netherlands; d Department of Pediatric Cardiac Surgery, Leiden University Medical Center, Leiden, the Netherlands

**Keywords:** Transposition of the great arteries, Arterial switch operation, Cardiac function, Tissue Doppler imaging, Speckle tracking echocardiography

## Abstract

**Background:**

Advanced echocardiographic techniques such as Tissue Doppler imaging (TDI) and speckle tracking echocardiography (STE) can detect more subtle changes in ventricular performance. We aimed to study the ventricular performance in patients with transposition of the great arteries (TGA) at mid-term follow-up after the arterial switch operation (ASO) with advanced echocardiographic techniques. In addition, we sought to discover new clinical phenotypes using unsupervised machine learning.

**Methods:**

Conventional, TDI and STE echocardiographic parameters were prospectively obtained from 124 TGA patients (66.1 % male, age 10.8 ± 5.1 years, 24.2 % with ventricular septal defect) in this observational study. The data was analyzed with conventional statistics and new machine learning techniques.

**Results:**

TGA patients had reduced biventricular systolic (septal s’ Z-score −2.28 ± 1.26; RV s’ Z-score −2.16 ± 0.71; mean left ventricular longitudinal strain Z-score of the LV -2.49 ± 1.68) and RV diastolic performance (RV E/e’ Z-score 2.35 ± 1.70) mid-term after ASO. Unsupervised clustering within the TGA population revealed 3 clusters. Interestingly, cluster 3 defined a group of patients with older age at ASO, the most reduced ventricular performance as well as the highest rates of reoperations and interventions.

**Conclusions:**

Assessment of ventricular performance with TDI and STE 10 years after ASO showed that TGA patients have decreased biventricular systolic and diastolic function, especially at the septal regions. Novel analytical methods such as unsupervised clustering may help identify new clinical phenotypes from multiple variables and may contribute to improved risk stratification.

## 1 Introduction

The arterial switch operation (ASO) is the gold standard for transposition of the great arteries (TGA) repair due to its low mortality and excellent outcomes [1,2]. However, TGA patients still face elevated risks of mortality and reintervention [[2], [3], [4]]. Indications for reintervention include right ventricular outflow tract obstruction, neo-aortic regurgitation and coronary artery abnormalities [2,3]. These factors may contribute to impairment of the right ventricular function, the left ventricular function, or both, over the medium to long term, contributing to increased long-term morbidity and mortality.

With tissue Doppler imaging (TDI) and speckle tracking echocardiography (STE) more subtle changes in ventricular performance can be detected than with conventional echocardiographic techniques [5,6]. This has also been demonstrated in the population with TGA after ASO, however, these previous studies included smaller sample sizes and used conventional analytical methods [[7], [8], [9], [10]]. The new analytical methods, like unsupervised machine learning, can be used for exploratory data analysis and pattern discovery within high-dimensional medical datasets.

The purpose of the present study was to investigate the ventricular performance based on TDI and STE in TGA patients at mid-term follow-up after ASO. Additionally, harnessing the power of unsupervised machine learning, we aimed to discover patterns in the distribution of baseline characteristics and the different echocardiographic parameters that may correlate with clinically significant phenotypes.

## 2 Materials and methods

### 2.1 Study population

Only simple TGA patients with and without ventricular septal defect (VSD) that underwent an arterial switch operation (ASO) were prospectively enrolled in this study. In all participants the ASO was conducted at the Leiden University Medical Center (LUMC) between 1993 and 2019 with cardiovascular follow-up of at least 2 years after ASO at one of two academic medical centers (LUMC and Amsterdam University Medical Center, the Netherlands). Patients with double outlet right ventricle and sub-pulmonary VSD (i.e. Taussig Bing anomaly) were excluded from the study. Transthoracic echocardiographic studies performed were part of a routine follow-up scheme of these patients and individual informed consent was waived. The studies were conducted in between February 2011 and March 2018. Hospital and outpatient records were reviewed to obtain demographics, preoperative anatomy and to collect clinical events. The study protocol conforms to the ethical guidelines of the 1975 Declaration of Helsinki as reflected in a priori approval by the institutional review board.

### 2.2 Echocardiographic measurements

Echocardiography data were acquired using a commercially available ultrasound system (Vivid E90 and E95, GE Vingmed Ultrasound, AS, Horten, Norway) and images were stored in digital format and analyzed off-line using EchoPac version 11.3 (General Electric Vingmed). Image acquisition and measurements were performed according to the recommendations of the American Society of Echocardiography [11,12] and previous work from our research group [13]. For the acquisition of the TDI images an adjusted sector width and angle to align the ultrasound beam along the direction of the myocardial motion was performed with a color frame rate of ≥120 frames/s. Standard 2-dimensional (2-D) grey-scale images (with optimized sector width and frame rate of 60–90 frames/second) were acquired from the apical views and digitally stored in cine-loop format to perform the STE analysis afterwards. All patients were in sinus rhythm with stable heart rate at the time of the study.

Two-dimensional grey-scale images, spectral Doppler, TDI tracings and STE analysis were performed from the apical 4-chamber (A4C) view. Left ventricular end-diastolic and end-systolic internal diameters (LVEDd and LVEDs) were assessed from M-mode recordings and fractional shortening (FS) was calculated: ([LVEDd−LVIDs]/LVEDd) × 100 %. The tricuspid annular plane systolic excursion (TAPSE) was measured with M-mode recordings from the A4C view. Z-scores for LVEDd, LVEDs [14], FS [15] and TAPSE [15] were calculated accordingly. Using the A4C view, ejection fraction (EF) was measured with the Simpson's biplane method. An EF >55 % was considered normal [16]. From pulsed-wave Doppler inflow tracings recorded in the A4C view the early-diastolic peak inflow velocity (E) and peak atrial contraction wave velocity (A) were determined. Additionally, the E/A ratio and the Z-scores for each parameter were calculated [17,18].

Assessment of branch pulmonary artery stenosis was performed with continuous-wave Doppler flow and severity was graded as: [0] normal (Vmax<2.0 m/s); [1] mild (Vmax = 2–3 m/s; [2] moderate (Vmax = 3–4 m/s); or [3] severe (Vmax>4 m/s). Neo-aortic valve regurgitation (neo-AR) was graded semi-quantitatively from the parasternal long axis by color Doppler imaging according to the international guidelines [19] and categorized as follows: [0] absent; [1] mild; or [2] moderate or severe.

The TDI longitudinal myocardial velocity curves of the basal LV lateral wall, basal septum, and basal RV lateral wall were obtained in the A4C view. Subsequently, peak systolic velocities (s’) and peak early (e’) and late diastolic velocities (a’) and the ratio between the E and the e’ of both LV and RV (E/e’ ratio) were calculated. In addition, the Z-scores of the RV and LV TDI parameters were calculated [17,18].

Speckle tracking echocardiography was performed in the two-dimensional grey-scale images from the LV A4C view (and 3 and 2 chamber views) as previously described by our group [13]. After tracing of the endocardial border of the LV, the LV was divided in 6 equal segments. In each segment tracking quality was automatically evaluated and this resulted in automatic rejection or acceptation of the segment. Although the observer could override this automatic decision based on visual evaluation [20], this was used very conservatively and regarded as feasible when at least five segments were scored as adequate. For this study, only the A4C view was included, as these images generally adhered to protocol standards, with 94 % of all patients meeting the inclusion criteria. Data obtained was displayed in 6 longitudinal time-strain curves for each segment of the LV (basal septal, mid-septal, apical septal, basal lateral, mid lateral and apical lateral) and the peak longitudinal strains (LS) and mean LS was obtained. Peak LS was defined as the most negative strain value at any time point during one cardiac cycle; Mean LS was defined as the average of all peak LS values of the individual segmental curves. Z-scores were calculated from pediatric reference values [21], except for the apical lateral segment, for which no Z-score equation was available.

Anthropometric, surgical and echocardiographic data, along with clinical factors, were analyzed for their association with ventricular performance. This analysis included age and BMI at the time of the study, age and weight at ASO, one- or two-stage surgical repair, coronary artery anatomy (according to the Leiden Convention coronary coding system) [22], the need for reoperation/reintervention post-ASO, and the grades of neo-AR and pulmonary artery stenosis.

### 2.3 Statistical analyses

Continuous variables are reported as mean ± standard deviation, while categorical variables are reported as frequencies and percentages. Variables were tested for normality by Shapiro-Wilk test. Continuous baseline characteristics were compared using one-way analysis of variance (ANOVA) with Tukey's post hoc correction for pairwise comparisons in case of normally distributed variables, or using Kruskal-Wallis test with Dunn's post hoc correction for pairwise comparisons in case of non-normally distributed variables. Categorical baseline characteristics were compared using Chi-squared test or Fisher's exact test, as appropriate, applying Bonferroni post hoc correction for comparisons of 2 groups. Since echocardiographic parameters are subject to developmental changes over ages in childhood, Z-scores were used for all parameters whenever applicable; the raw values for each parameter are given in Supplemental Tables S1 and S2.

While the first sets of analyses examined each parameter one at a time, we next sought to discover patterns in the distribution of baseline characteristics as well as conventional echocardiography, TDI and STE parameters within the TGA population. To achieve this goal, two machine learning techniques were applied, including principal component analysis (PCA) and k-means clustering. First, patterns were identified using PCA, which is a technique for reducing the dimensionality of multivariable datasets, with the aim of increasing interpretability with a minimal loss of information. It does so by creating new uncorrelated variables, referred to as the Principal Components (PC), which are defined by the dataset, not a priori. Second, k-means clustering was performed, which is a method that aims to partition n observations into k clusters in which each observation belongs to the cluster with the nearest mean (referred to as the cluster centroid). The ideal number of clusters was determined using the elbow method, which determines the number of clusters beyond which adding another cluster does not improve much on the total within-cluster sum of square. Both steps were implemented using the R package “factoextra” (version 1.0.7). All characteristics presented in Table 1, Table 2 were considered for these analyses. Missing data were imputed using a random forest model via the R package “missForest” (version 1.4) prior to performing these analyses. Variables with high multicollinearity were filtered out based on correlation analysis (Supplemental Fig. S1). All analyses were completed with R Statistical Software (version 4.1.1, Foundation for Statistical Computing, Vienna, Austria). The complete code is available from the corresponding author upon reasonable request.

**Table 1:** Baseline characteristics and echocardiographic parameters in the study population.

Variable	All patients (n = 124)	TGA-IVS (n = 94)	TGA-VSD (n = 30)	P-value
**Patient characteristics**				
Male sex	82 (66.1 %)	63 (67.0 %)	19 (63.3 %)	0.881
Age at echo, years	10.8 ± 5.1	10.6 ± 4.9	11.5 ± 5.6	0.435
BMI at echo, kg/m^2^	18.5 ± 4.0	18.5 ± 4.0	18.4 ± 3.9	0.937
Age at ASO, days	16.0 ± 22.3	13.1 ± 19.7	25.0 ± 27.3	**0.033**
Weight at ASO, kg	3.5 ± 0.6	3.40 ± 0.6	3.7 ± 0.6	**0.027**
Two-stage ASO	6 (4.8 %)	4 (4.3 %)	2 (6.67 %)	0.631
Usual coronary anatomy (1LCx-2R)^a^	78 (62.9 %)	62 (66.0 %)	16 (53.3 %)	0.303
**Follow-up characteristics**				
Any reoperation	19 (15.3 %)	10 (10.6 %)	9 (30.0 %)	**0.018**
Any intervention	16 (12.9 %)	10 (10.6 %)	6 (20.0 %)	0.214
Highest pulmonary artery gradient, mmHg	26.4 ± 14.9	26.7 ± 14.7	25.5 ± 15.5	0.760
Pulmonary artery stenosis grade				0.982
0	24 (19.4 %)	18 (19.1 %)	6 (20.0 %)	
1	76 (61.3 %)	58 (61.7 %)	19 (63.3 %)	
2	18 (14.5 %)	13 (13.8 %)	4 (13.3 %)	
3	6 (4.84 %)	5 (5.32 %)	1 (3.33 %)	
Neo-aortic valve regurgitation grade				0.602
0	86 (69.4 %)	67 (71.3 %)	19 (63.3 %)	
1	32 (25.8 %)	23 (24.5 %)	9 (30.0 %)	
2	6 (4.8 %)	4 (4.3 %)	2 (6.7 %)	
**Conventional parameters**				
LVEDd Z-score	1.17 ± 1.22	1.12 ± 1.25	1.30 ± 1.12	0.465
LVEDs Z-score	0.84 ± 1.12	0.80 ± 1.15	0.97 ± 1.03	0.467
FS, %	36.5 ± 3.9	36.7 ± 3.86	36.0 ± 4.13	0.451
HR, bpm	75.1 ± 20.4	75.4 ± 20.5	74.1 ± 20.4	0.771
LVEF, %	51.6 ± 3.93	51.7 ± 3.6	51.1 ± 4.78	0.522
TAPSE Z-score	−2.92 ± 1.93	−2.76 ± 1.87	−3.42 ± 2.04	0.113
MV E velocity Z-score	0.55 ± 1.24	0.67 ± 1.11	0.16 ± 1.55	0.103
MV A velocity Z-score	0.08 ± 1.24	0.07 ± 1.22	0.08 ± 1.34	0.882
MV E/A ratio Z-score	0.18 ± 1.25	0.26 ± 1.23	−0.05 ± 1.32	0.229
TV E velocity Z-score	1.32 ± 1.31	1.20 ± 1.14	1.61 ± 1.70	0.222
TV A velocity Z-score	0.46 ± 1.15	0.31 ± 1.12	0.92 ± 1.16	**0.016**
TV E/A ratio Z-score	0.43 ± 0.96	0.61 ± 1.02	0.21 ± 0.91	**0.033**
**TDI parameters**				
LV s' velocity Z-score	−0.87 ± 1.14	−0.95 ± 1.02	−0.66 ± 1.45	0.342
LV e' velocity Z-score	−0.15 ± 1.19	−0.18 ± 1.23	−0.08 ± 1.09	0.681
LV a' velocity Z-score	−0.73 ± 1.26	−0.82 ± 1.28	−0.42 ± 1.16	0.109
Septal s' velocity Z-score	−2.28 ± 1.26	−2.20 ± 1.28	−2.54 ± 1.16	0.182
Septal e' velocity Z-score	−1.04 ± 1.00	−0.92 ± 0.96	−1.43 ± 1.03	0.021
Septal a' velocity Z-score	−0.52 ± 1.12	−0.43 ± 1.09	−0.79 ± 1.18	0.154
RV s' velocity Z-score	−2.16 ± 0.71	−2.09 ± 0.72	−2.38 ± 0.61	**0.034**
RV e' velocity Z-score	−1.16 ± 1.04	−1.04 ± 1.03	−1.54 ± 1.00	**0.023**
RV a' velocity Z-score	−1.53 ± 0.52	−1.50 ± 0.53	−1.59 ± 0.49	0.430
LV E/e' ratio Z-score	0.70 ± 1.38	0.85 ± 1.27	0.29 ± 1.61	0.096
RV E/e' ratio Z-score	2.35 ± 1.70	2.18 ± 1.68	2.90 ± 1.72	**0.048**
**STE parameters of LV**				
Basal septal LS Z-score	−1.41 ± 1.30	−1.32 ± 1.28	−1.70 ± 1.34	0.164
Mid septal LS Z-score	−0.81 ± 1.32	−0.71 ± 1.35	−1.14 ± 1.17	0.097
Apical septal LS Z-score	−1.31 ± 1.31	−1.22 ± 1.34	−1.61 ± 1.17	0.117
Apical lateral LS Z-score	NA	NA	NA	NA
Mid lateral LS Z-score	−1.24 ± 1.57	−1.30 ± 1.49	−1.01 ± 1.83	0.443
Basal lateral LS Z-score	−0.10 ± 1.09	−0.11 ± 1.13	−0.08 ± 0.94	0.935
Mean LS (4-chamber) Z-score	−2.49 ± 1.68	−2.40 ± 1.56	−2.78 ± 2.00	0.352

*ASO*, arterial switch operation; *BMI*, body mass index; *FS*, fractional shortening; *HR*, heart rate; *IVS*, intact ventricular septum; *LS*, longitudinal strain; *LV*, left ventricle/ventricular; *LVEDd*, left ventricular end-diastolic dimension; *LVEDs*, left ventricular end-systolic dimension; *LVEF*, left ventricular ejection fraction; *MV*, mitral valve; *NA*, not applicable; *RV*, right ventricle/ventricular; *STE*, speckle tracking echocardiography; *TAPSE*, tricuspid annular plane systolic excursion; *TDI*, tissue Doppler imaging; *TGA*, transposition of great arteries; *TV*, tricuspid valve; *VSD*, ventricular septal defect.

a According to the Leiden Convention coronary coding system.

**Table 2:** Baseline characteristics and echocardiographic parameters of the clusters within the TGA population.

Variable	Cluster 1 (n = 37)	Cluster 2 (n = 53)	Cluster 3 (n = 34)	P-value overall	P-value 1 vs 2	P-value 1 vs 3	P-value 2 vs 3
**Patient characteristics**							
Male sex	20 (54.1 %)	36 (67.9 %)	26 (76.5 %)	0.128	0.398	0.253	0.537
Age at echo, years	13.1 ± 3.89	6.71 ± 3.78	14.9 ± 2.82	**<0.001**	**<0.001**	0.081	**<0.001**
BMI at echo, kg/m^2^	19.5 ± 3.57	16.6 ± 2.89	20.3 ± 4.59	**<0.001**	**0.001**	0.574	**<0.001**
Age at ASO, days	16.5 ± 24.8	10.8 ± 6.20	23.4 ± 31.9	**0.036**	0.453	0.382	**0.027**
Weight at ASO, kg	3.53 ± 0.74	3.34 ± 0.40	3.58 ± 0.60	0.111	0.260	0.931	0.136
Two-stage ASO	3 (8.11 %)	0 (0.0 %)	3 (8.82 %)	**0.046**	0.099	1.000	0.099
Usual coronary anatomy (1LCx-2R)^a^	24 (64.9 %)	33 (62.3 %)	21 (61.8 %)	0.956	1.000	1.000	1.000
Ventricular septal defect	6 (16.2 %)	11 (20.8 %)	13 (38.2 %)	0.071	0.789	0.187	0.187
**Follow-up characteristics**							
Any reoperation	3 (8.11 %)	3 (5.66 %)	13 (38.2 %)	**<0.001**	0.687	**0.009**	**0.001**
Any intervention	5 (13.5 %)	0 (0.0 %)	11 (32.4 %)	**<0.001**	**0.015**	0.107	**<0.001**
Highest pulmonary artery gradient, mmHg	28.9 ± 14.5	21.4 ± 11.5	31.6 ± 17.7	**0.003**	**0.044**	0.703	**0.005**
Pulmonary artery stenosis grade				0.060	0.136	0.413	0.126
0	4 (10.8 %)	15 (28.3 %)	5 (14.7 %)				
1	25 (67.6 %)	33 (62.3 %)	18 (52.9 %)				
2	7 (18.9 %)	4 (7.55 %)	7 (20.6 %)				
3	1 (2.70 %)	1 (1.89 %)	4 (11.8 %)				
Neo-aortic valve regurgitation grade				**0.020**	0.087	0.420	**0.023**
0	24 (64.9 %)	44 (83.0 %)	18 (52.9 %)				
1	12 (32.4 %)	7 (13.2 %)	13 (38.2 %)				
2	1 (2.70 %)	2 (3.77 %)	3 (8.82 %)				
**Conventional echocardiography parameters**							
LVEDd Z-score	1.35 ± 1.25	0.94 ± 1.11	1.32 ± 1.32	0.206	0.264	0.994	0.336
LVEDs Z-score	0.70 ± 1.06	0.87 ± 1.07	0.96 ± 1.25	0.614	0.764	0.601	0.931
FS, %	37.9 ± 3.92	35.8 ± 3.32	36.2 ± 4.47	**0.035**	**0.032**	0.141	0.907
HR, bpm	71.5 ± 19.0	83.3 ± 22.4	66.1 ± 12.8	**<0.001**	**0.013**	0.476	**<0.001**
LVEF, %	54.3 ± 3.72	50.3 ± 2.92	50.6 ± 4.15	**<0.001**	**<0.001**	**<0.001**	0.929
TAPSE Z-score	−2.38 ± 1.97	−2.80 ± 1.88	−3.68 ± 1.76	**0.014**	0.551	**0.012**	0.089
MV E velocity Z-score	1.00 ± 1.28	0.20 ± 1.18	0.60 ± 1.14	**0.009**	**0.007**	0.360	0.273
MV A velocity Z-score	0.47 ± 1.45	−0.37 ± 1.07	0.35 ± 1.04	**0.002**	**0.004**	0.902	**0.019**
MV E/A ratio Z-score	0.08 ± 1.46	0.37 ± 1.16	−0.01 ± 1.14	0.341	0.539	0.951	0.365
TV E velocity Z-score	1.49 ± 1.29	1.07 ± 1.07	1.53 ± 1.61	0.183	0.293	0.992	0.250
TV A velocity Z-score	0.55 ± 1.41	0.32 ± 0.93	0.58 ± 1.16	0.525	0.641	0.993	0.579
TV E/A ratio Z-score	0.54 ± 1.10	0.47 ± 0.84	0.26 ± 0.98	0.432	0.930	0.426	0.580
**TDI parameters**							
LV s' velocity Z-score	−0.74 ± 1.09	−0.71 ± 0.87	−1.27 ± 1.47	0.057	0.989	0.123	0.063
LV e' velocity Z-score	0.01 ± 1.24	−0.21 ± 1.13	−0.23 ± 1.25	0.614	0.659	0.666	0.996
LV a' velocity Z-score	−0.33 ± 1.13	−1.06 ± 1.20	−0.66 ± 1.36	**0.022**	**0.017**	0.493	0.301
Septal s' velocity Z-score	−1.16 ± 1.08	−2.65 ± 0.89	−2.93 ± 1.16	**<0.001**	**<0.001**	**<0.001**	0.425
Septal e' velocity Z-score	−0.68 ± 0.82	−0.91 ± 0.93	−1.65 ± 1.03	**<0.001**	0.498	**<0.001**	**0.001**
Septal a' velocity Z-score	0.21 ± 0.96	−0.91 ± 0.94	−0.70 ± 1.18	**<0.001**	**<0.001**	**0.001**	0.625
RV s' velocity Z-score	−0.86 ± 1.17	−1.00 ± 0.79	−1.73 ± 1.03	**<0.001**	0.783	**0.001**	**0.002**
RV e' velocity Z-score	−1.26 ± 0.56	−1.60 ± 0.44	−1.71 ± 0.46	**<0.001**	**0.003**	**<0.001**	0.600
RV a' velocity Z-score	−1.54 ± 0.63	−2.42 ± 0.54	−2.42 ± 0.60	**<0.001**	**<0.001**	**<0.001**	1.000
LV E/e' ratio Z-score	1.02 ± 1.45	0.43 ± 1.39	0.79 ± 1.23	0.123	0.113	0.766	0.448
RV E/e' ratio Z-score	1.98 ± 1.52	2.14 ± 1.51	3.07 ± 1.96	**0.012**	0.894	**0.017**	**0.030**
**STE parameters of LV**							
Basal septal LS Z-score	−0.93 ± 1.18	−1.08 ± 1.06	−2.47 ± 1.18	**<0.001**	0.824	**<0.001**	**<0.001**
Mid septal LS Z-score	−0.19 ± 1.14	−0.68 ± 0.94	−1.69 ± 1.55	**<0.001**	0.139	**<0.001**	**0.001**
Apical septal LS Z-score	−0.55 ± 1.08	−1.63 ± 1.11	−1.65 ± 1.49	**<0.001**	**<0.001**	**0.001**	0.995
Apical lateral LS Z-score	NA	NA	NA	NA	NA	NA	NA
Mid lateral LS Z-score	−0.76 ± 1.57	−0.86 ± 1.27	−2.34 ± 1.50	**<0.001**	0.938	**<0.001**	**<0.001**
Basal lateral LS Z-score	0.05 ± 1.24	0.09 ± 0.96	−0.55 ± 0.99	**0.015**	0.986	**0.046**	**0.018**
Mean LS (4-chamber) Z-score	−1.53 ± 1.36	−2.22 ± 1.14	−3.98 ± 1.73	**<0.001**	0.058	**<0.001**	**<0.001**

*ASO*, arterial switch operation; *BMI*, body mass index; *FS*, fractional shortening; *HR*, heart rate; *LS*, longitudinal strain; *LV*, left ventricle/ventricular; *LVEDd*, left ventricular end-diastolic dimension; *LVEDs*, left ventricular end-systolic dimension; *LVEF*, left ventricular ejection fraction; *MV*, mitral valve; *RV*, right ventricle/ventricular; NA, not applicable; *STE*, speckle tracking echocardiography; *TAPSE*, tricuspid annular plane systolic excursion; *TDI*, tissue Doppler imaging; *TV*, tricuspid valve.

a According to the Leiden Convention coronary coding system.

## 3 Results

### 3.1 Study population

A total of 124 TGA patients (66.1 % male, age 10.8 ± 5.1 years) were enrolled in this study. Ninety-four patients had TGA-IVS (75.8 %) and 30 patients had TGA with VSD (24.4 %). Six patients underwent a two-stage ASO procedure, involving pulmonary artery banding and/or modified Blalock–Thomas-Taussig shunt as initial step prior to ASO, due to delayed presentation (n = 4), anatomical complexities (n = 1, aneurysmatic membranous septum leading to left ventricular outflow tract obstruction) or neonatal conditions requiring pre-ASO stabilization (n = 1, cerebrovascular event). Baseline characteristics are summarized in Table 1. In the TGA-VSD group, the age at surgery was significantly higher compared to the TGA-IVS patients (TGA-VSD 25 days vs the TGA-IVS 13 days, p = 0.033). Usual coronary anatomy (1LCx-2R) was observed in 62.9 %, 15.3 % had a 1L-2CxR anatomy, 6,5 % 1RL-2Cx, 4 % 1L2RnoCx 3.2 % 2LCxR, 1.6 % 1R-2LCx and in 4 % the anatomy was unknown. Reoperations occurred in 15.3 % and percutaneous interventions in 12.9 % of the patients. At least moderate pulmonary stenosis was present in 24 patients (19.3 %), and at least moderate neo-AR in 6 patients (4.8 %).

### 3.2 Ventricular performance–conventional analysis

Left ventricular and RV echocardiographic parameters used to determine ventricular performance in the TGA cohort (expressed in Z-scores) are depicted in Table 1 and Table S1 (raw data). For the STI analysis, 94 % of patients were deemed to have good-quality images. Regarding TDI, 95 % of patients were eligible for LV wall assessment, 98 % for the septum, and 97 % for RV assessment.

*LV systolic function*. Both FS (36.5 ± 3.9 %) and LVEF (51.6 ± 3.93 %) were within the normal reference values for children. However, the mean LS Z-score was relatively low (−2.49 ± 1.68) suggesting a diminished LV systolic function. The septal part of the LV was the most affected, as reflected by lower septal s’ velocity Z-score (−2.28 ± 1.26).

*LV diastolic function*. The LV and septal e’ velocities (LV e’ Z-score −0.15 ± 1.19; septal e’ Z-score −1.04 ± 1.00) and the LV E/e’ ratio Z-scores (0.70 ± 1.38) were within the normal ranges.

*RV systolic function*. The systolic RV function was diminished reflected by TAPSE Z-score (−2.92 ± 1.93) and RV s’ velocity Z-score (−2.16 ± 0.71).

*RV diastolic function*. The tricuspid valve E/A ratio was preserved but the RV E/e’ ratio Z-score (2.35 ± 1.70) was above the normal range, suggesting reduced RV diastolic performance.

Patients with VSD had a higher TV A velocity Z-score and therefore lower TV E/A ratio Z-score than those without VSD. Besides, TGA-VSD was associated with lower RV s’ and e’ velocity Z-scores and higher RV E/e’ ratio Z-score when compared to TGA-IVS (Table 1).

### 3.3 Ventricular performance–unsupervised clustering analysis

To agnostically detect patterns in the distribution of baseline characteristics as well as conventional echocardiography, TDI and STE parameters among all TGA patients, an unsupervised clustering algorithm was applied. In the first set of analyses, PCA revealed a two-factor solution that accounted for 21.8 % of the variability in the data (Fig. 1A). The most important contributors to PC1 were age at echo, septal a’, and RV s’ velocity whereas mean strain, RV s’ velocity, and septal s’ velocity contributed most to PC 2 (Fig. 1B–C). Subsequently, k-means clustering identified 3 distinct clusters (Fig. 1D–E, Table 2, Table S2).

**Fig. 1:**
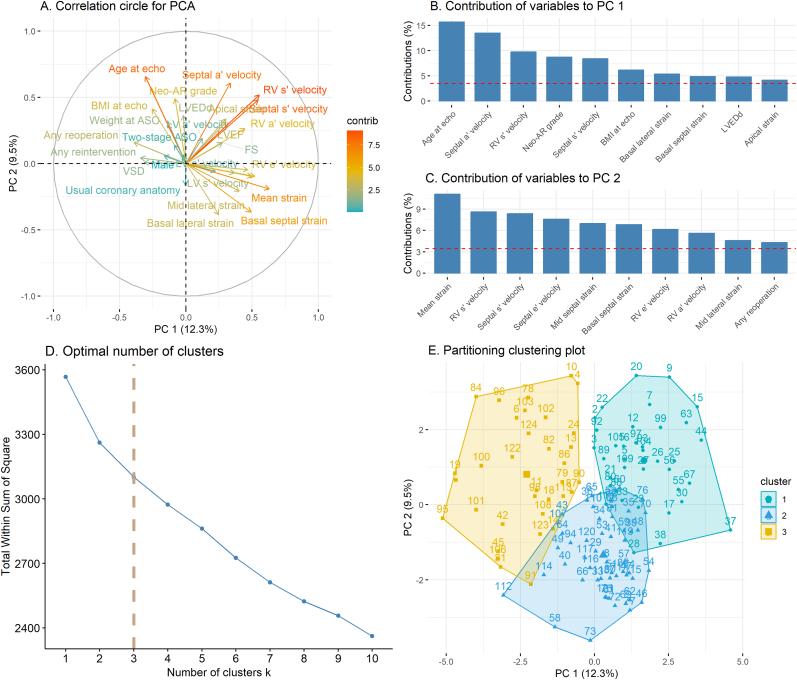
**Unsupervised clustering to detect patterns in baseline characteristics as well as conventional echocardiography, TDI and STE parameters among TGA patients. (A)** Correlation circle displaying the original variables as vectors in a 2-dimensional space created by the two principal components (PC). The length and color of the vector represent the overall contribution of the variable, while the projection of the vector onto one of the PCs gives the contribution to that specific PC. The contributions are based on the cosine squared. **(B, C)** Contributions of variables to PC 1 and PC 2. The variables are ranked according to their contribution to each PC. The red dashed lines on the graphs on the right indicate the expected average contribution; for a given component, a variable with a contribution larger than this cutoff could be considered important in contributing to the component. **(D)** Total within-cluster sum of squares for various numbers of clusters. Based on the elbow method, the optimal number of clusters was determined to be three. **(E)** Partitioning clustering plot, with all patients positioned in a cartesian system based on the two PCs and colored according to the cluster to which they belonged based on k-means clustering. *ASO*, arterial switch operation; *FS,* fractional shortening; *LV*, left ventricle/ventricular; *LVEDd*, left ventricular end-diastolic dimension; *LVEF*, left ventricular ejection fraction; *PC*, principal component; *PCA*, principal component analysis; *RV*, right ventricle/ventricular; *STE*, speckle tracking echocardiography; *TDI*, tissue Doppler imaging; *TGA*, transposition of the great arteries; *VSD*, ventricular septal defect.

Differences in baseline characteristics and in ventricular performance parameters between the 3 clusters are depicted in Table 2. The most relevant finding was that patients in cluster 3 (n = 34, 27.4 %) had overall the worst ventricular performance parameters. This group had significantly the lowest Z-scores for mean LS (−3.98 ± 1.73), basal septal LS, basal lateral LS, mid septal LS, and mid lateral LS; the lowest RV s’ velocity Z-score (−1.73 ± 1.03); the lowest septal e’ velocity Z-score (−1.65 ± 1.03); and the highest RV E/e’ ratio Z-score (3.07 ± 1.96) (Table 2). This cluster consisted of patients who underwent surgery at older ages and had the highest rates of reoperation and reintervention after ASO. Moreover, they presented the highest pulmonary artery gradients and neo-aortic valve regurgitation grades (Table 2).

## 4 Discussion

To our knowledge, this is the largest study on ventricular performance using advanced echocardiographic techniques in TGA patients mid-term post-ASO. Moreover, we employed unsupervised clustering to identify clinical phenotypes from baseline and advanced echocardiographic data. Key findings include: (i) advanced echocardiographic techniques (TDI and STE) showed reduced LV systolic and RV systolic/diastolic performance despite preserved fractional shortening, with more pronounced reduction in RV function in the TGA-VSD group; (ii) unsupervised clustering identified a sub-phenotype with older age at ASO, higher reoperation/reintervention rates, more significantly impaired ventricular performance, and a tendency towards higher pulmonary artery gradients and higher neo-aortic valve regurgitation grades.

### 4.1 Left ventricle

Previous studies that evaluate LV performance in TGA patients after ASO showed generally a preserved LV EF and/or FS [7,[23], [24], [25]]. The LV FS was also normal in our cohort, but the EF was slightly diminished compared to the normal values [16]. As previously mentioned, new echocardiographic techniques have proven to detect more subtle changes in ventricular impairment than the classic techniques in different congenital heart defects and they allow us to detect regional differences [6,26]. In this study, the Z-scores of the mean LS of the LV and the septal s’ velocity were lower than normal, suggesting lower LV systolic performance, especially at the septal level.

It has been described that LV function is diminished shortly after ASO and seems to recover to normal within the first year after surgery [25]. However, using unsupervised clustering we found a trend towards more reduced systolic LV performance parameters and older patients at the time of the echo. In the same line, other groups have reported lower LS in TGA patients during further follow-up including adults [7,24,27]. Notably, no differences in LV performance between patients with or without VSD were observed.

The mechanisms behind reduced LV deformation remains unclear. Longitudinal strain reduction has been related to ischemia from reduced coronary perfusion (due to anatomic abnormalities, reduced flow reserve or abnormal vasomotor response) [27]. Longitudinally orientated endocardial fibers, vulnerable to hypoperfusion and fibrotic remodeling, may exhibit reduced LS as an initial sign of declining ventricular function [27]. Most TGA patients in our cohort had a typical coronary anatomy (1LCx-2R), yet it didn't affect ventricular performance in our clustering analysis. A previous magnetic resonance imaging (MRI) study in children after ASO found no myocardial scarring or perfusion defects, but noted signs of ventricular and myocardial remodeling, including increased LV dimensions and fibrosis indicators [7]. Additionally, postmortem examination of TGA specimens revealed neonatal hearts with diffuse interstitial fibrosis, suggesting that alterations in oxygen saturation may affect myocardial structure prenatally [28]. Previous studies have demonstrated a significant correlation between age at surgery and global LS reduction, suggesting that older age might lead to greater loss of myocardial mass and a more hypoplastic left anterior descending coronary artery [29]. Similarly, in our study the cluster with older age at ASO exhibited lower mean LS and LV s’ velocity.

Possible causes of LV diastolic dysfunction in TGA patients include delayed relaxation, impaired LV filling pressure due to fibrotic remodeling from ischemia or increased aortic stiffness leading to LV hypertrophy [7,30]. In our study, LV diastolic function was observed to be normal, which may indicate that the degree of myocardial remodeling or stiffness in our cohort was not sufficient to affect diastolic performance. Grotenhuis et al., identified diffuse myocardial fibrosis in TGA patients post-ASO but found no strong association with LV diastolic dysfunction diastolic parameters, however although the LV diastolic parameters were slightly worse compared to healthy controls, they remained within normal ranges. Including CMR imaging to assess diffuse myocardial fibrosis and evaluating aortic stiffness would have provided valuable insights into their potential impact on LV diastolic function.

### 4.2 Right ventricle

Right ventricular performance in TGA patients post-ASO remains poorly understood. In our study, TGA patients showed a diminished RV systolic and diastolic function, with lower TAPSE and RV s’ Z-scores and higher RV E/e’ ratio Z-score. Impairment of the RV, assessed by TDI parameters, has prognostic significance in heart failure patients [31]. Klitsie et al. also found reduced systolic and diastolic RV performance post-ASO, persisting at mid-term follow-up, consistent with other smaller cohort studies [25,27,32]. Of note, RV impairment was more pronounced in the TGA-VSD subgroup. This might be related to abnormal septal movement after VSD closure, while even after isolated VSD closure RV function may be influenced on long-term. Furthermore, the TGA-IVS patients were operated on earlier than the TGA-VSD patients which might have influenced long-term RV function.

In unsupervised clustering, the cluster with the oldest age at ASO and highest reoperation/reintervention rates had the lowest RV performance parameters (lower RVs' and higher RV E/e’ ratio Z-scores). Possible causes of RV impairment include preoperative ischemia and hypoxia, as older patients at surgery endure prolonged exposure to hypoxia and systemic pressure. Other factors contributing to RV dysfunction post-surgery may include incomplete or uneven myocardial protection, cardiopulmonary bypass use, or residual pulmonary artery (branch) stenosis [33].

Pulmonary artery (branch) stenosis, often present after ASO [[2], [3], [4]], elevates RV afterload, potentially impairing RV performance. We noted that patients with higher pulmonary artery gradients tended to exhibit more reduced RV diastolic performance. Interestingly, in the study by Grotenhuis et al., TGA patients even without significant anatomical stenosis showed an increased peak flow velocity in the pulmonary artery, that was associated to RV hypertrophy and relaxation abnormalities while systolic function was preserved [32].

### 4.3 Unsupervised clustering

Unsupervised clustering analysis in TGA patients identified a vulnerable cluster of patients with more pronounced decrease in ventricular performance parameters.

Previous studies have highlighted the low operative mortality and excellent long-term survival of ASO, but concerns about reinterventions, especially for pulmonary stenosis and right-sided obstructions, remain [[2], [3], [4]]. Left-sided interventions, though less frequent, also occur [2,4,34]. In addition, although most patients are in NYHA class 1, exercise capacity is usually reduced (80%–85 % of normal) [35]. Prior studies have found correlations between reduced ventricular performance, exercise capacity, and pulmonary stenosis [35]. However, multivariable analysis does not fully identify subgroups at higher risk for future interventions. Unsupervised clustering, used in other congenital heart defect studies, can reliably identify subgroups at-risk [36]. Despite the absence of exercise and MRI data in this study, we identified a subgroup characterized by older age at surgery, lower biventricular function and more frequent reinterventions. This subgroup also appeared to have higher pulmonary artery gradients, and more severe neo-aortic valve regurgitation grades. Although unsupervised clustering does not establish a cause-effect relationship, it may be hypothesized that older age at ASO could be a primary indicator of adverse outcomes, including RV dysfunction and increased risk of reintervention.

## 5 Limitations

This prospective study used echocardiograms to follow TGA patients post-ASO. One limitation of this study was the exclusive use of the apical 4-chamber view for STE analysis, potentially limiting a comprehensive assessment of myocardial performance. However, in healthy children the value of GLS based on a 6-segments model seems as good as that based on the 18-segments model [20].

Similarly, the evaluation of RV LS frequently lacked sufficient image quality data. Nonetheless, other RV function measurements were available and revealed changes in both diastolic and systolic function. The absence of exercise data and MRI parameters limited the detail of unsupervised clustering. Future research should include these parameters and cover a wider age range.

Another limitation of this study is the lack of external validation for the novel machine learning methods applied. While the findings provide promising insights, validation on independent datasets is necessary to confirm the robustness and generalizability of the approach.

## 6 Conclusions

Assessment of ventricular performance with TDI and STE showed that TGA patients 10 years after ASO have decreased biventricular systolic and diastolic function. These findings reinforce the need of a long-term follow-up of the ventricular performance in this group of patients with these echocardiographic techniques that detect more subtle and regional changes in ventricular function. In addition, the unsupervised cluster analysis revealed a subgroup of patients with older age at ASO and decreased biventricular function in combination with an increased risk of reinterventions linked to the presence of higher gradients in the pulmonary arteries as well as more severe neo-aortic valve regurgitation.

## CRediT authorship contribution statement

**Covadonga Terol Espinosa de los Monteros:** Writing – original draft, Methodology, Formal analysis, Data curation, Conceptualization. **Roel L.F. van der Palen:** Writing – review & editing, Supervision, Formal analysis, Data curation, Conceptualization. **Jef Van den Eynde:** Writing – original draft, Validation, Formal analysis. **Lukas Rammeloo:** Writing – review & editing, Validation. **Mark G. Hazekamp:** Writing – review & editing, Validation. **Nico A. Blom:** Writing – review & editing, Validation, Supervision, Conceptualization. **Irene M. Kuipers:** Writing – review & editing, Validation. **Arend D.J. ten Harkel:** Writing – review & editing, Validation, Supervision, Formal analysis, Conceptualization.

## Funding

This research did not receive any specific grant from funding agencies in the public, commercial, or not-for-profit sectors.

## Declaration of competing interest

The authors declare that they have no known competing financial interests or personal relationships that could have appeared to influence the work reported in this paper.

## Figures and Tables

**figs1:**
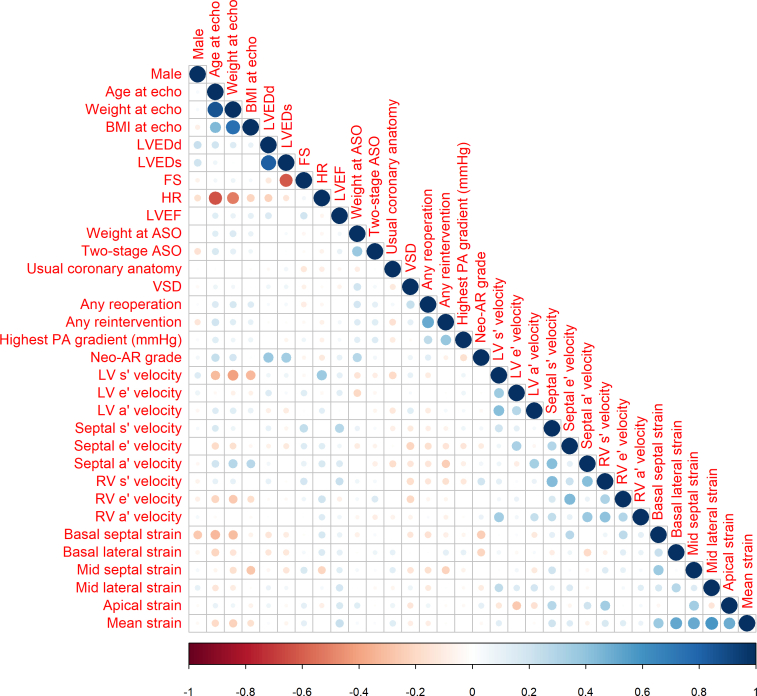


## References

[1] Sarris G.E.C.G., Balmer C.S., Bonou P.G. (2017). Clinical guidelines for the management of patients with transposition of the great arteries with intact ventricular septum. Eur J Cardio Thorac Surg.

[2] van der Palen R.L.F., Blom N.A., Kuipers I.M. (2021). Long-term outcome after the arterial switch operation: 43 years of experience. Eur J Cardio Thorac Surg.

[3] Fraser C.D., Chacon-Portillo M.A., Well A. (2020). Twenty-three-year experience with the arterial switch operation: expectations and long-term outcomes. Semin Thorac Cardiovasc Surg.

[4] Fricke T.A., Buratto E., Weintraub R.G. (2022). Long-term outcomes of the arterial switch operation. J Thorac Cardiovasc Surg.

[5] Voigt J.U., Cvijic M. (2019). 2- and 3-dimensional myocardial strain in cardiac health and disease. JACC Cardiovasc Imaging.

[6] Klitsie L.M., Roest A.A., Blom N.A. (2014). Ventricular performance after surgery for a congenital heart defect as assessed using advanced echocardiography: from Doppler flow to 3D echocardiography and speckle-tracking strain imaging. Pediatr Cardiol.

[7] Grotenhuis H.B., Cifra B., Mertens L.L. (2019). Left ventricular remodelling in long-term survivors after the arterial switch operation for transposition of the great arteries. Eur Heart J Cardiovasc Imaging.

[8] Bragantini G., Bartolacelli Y., Balducci A. (2022). Left ventricle function after arterial switch procedure for D-transposition of the great arteries: long term evaluation by speckle-tracking analysis. Int. J. Cardiol. Congen. Heart Dis..

[9] Wang C., Li V.W., So E.K. (2020). Left ventricular stiffness in adolescents and young adults after arterial switch operation for complete transposition of the great arteries. Pediatr Cardiol.

[10] Di Salvo G., Al Bulbul Z., Issa Z. (2016). Left ventricular mechanics after arterial switch operation: a speckle-tracking echocardiography study. J Cardiovasc Med.

[11] Lopez L., Colan S.D., Frommelt P.C. (2010). Recommendations for quantification methods during the performance of a pediatric echocardiogram: a report from the pediatric measurements writing group of the American society of echocardiography pediatric and congenital heart disease council. J Am Soc Echocardiogr.

[12] Mitchell C., Rahko P.S., Blauwet L.A. (2019). Guidelines for performing a comprehensive transthoracic echocardiographic examination in adults: recommendations from the American society of echocardiography. J Am Soc Echocardiogr.

[13] Terol C., Kamphuis V.P., Hazekamp M.G. (2020). Left and right ventricular impairment shortly after correction of tetralogy of fallot. Pediatr Cardiol.

[14] Kampmann C., Wiethoff C.M., Wenzel A. (2000). Normal values of M mode echocardiographic measurements of more than 2000 healthy infants and children in central Europe. Heart.

[15] Koestenberger M., Ravekes W., Everett A.D. (2009). Right ventricular function in infants, children and adolescents: reference values of the tricuspid annular plane systolic excursion (TAPSE) in 640 healthy patients and calculation of z score values. J Am Soc Echocardiogr.

[16] Diaz A., Zocalo Y., Bia D. (2019). Reference intervals and percentile curves of echocardiographic left ventricular mass, relative wall thickness and ejection fraction in healthy children and adolescents. Pediatr Cardiol.

[17] Dallaire F., Slorach C., Hui W. (2015). Reference values for pulse wave Doppler and tissue Doppler imaging in pediatric echocardiography. Circ Cardiovasc Imag..

[18] Eidem B.W., McMahon C.J., Cohen R.R. (2004). Impact of cardiac growth on Doppler tissue imaging velocities: a study in healthy children. J Am Soc Echocardiogr.

[19] Zoghbi W.A., Enriquez-Sarano M., Foster E. (2003). Recommendations for evaluation of the severity of native valvular regurgitation with two-dimensional and Doppler echocardiography. J Am Soc Echocardiogr.

[20] Klitsie L.M., Roest A.A., van der Hulst A.E. (2013). Assessment of intraventricular time differences in healthy children using two-dimensional speckle-tracking echocardiography. J Am Soc Echocardiogr.

[21] Dallaire F., Slorach C., Bradley T. (2016). Pediatric reference values and Z score equations for left ventricular systolic strain measured by two-dimensional speckle-tracking echocardiography. J Am Soc Echocardiogr.

[22] Gittenberger-de Groot A.C., Koenraadt W.M.C., Bartelings M.M. (2018). Coding of coronary arterial origin and branching in congenital heart disease: the modified Leiden Convention. J Thorac Cardiovasc Surg.

[23] van Wijk S.W., Driessen M.M., Meijboom F.J. (2018). Left ventricular function and exercise capacity after arterial switch operation for transposition of the great arteries: a systematic review and meta-analysis. Cardiol Young.

[24] van Wijk S.W., Driessen M.M.P., Meijboom F.J. (2019). Evaluation of left ventricular function long term after arterial switch operation for transposition of the great arteries. Pediatr Cardiol.

[25] Klitsie L.M., Roest A.A., Kuipers I.M. (2014). Left and right ventricular performance after arterial switch operation. J Thorac Cardiovasc Surg.

[26] Bruch C., Stypmann J., Grude M. (2004). Tissue Doppler imaging in patients with moderate to severe aortic valve stenosis: clinical usefulness and diagnostic accuracy. Am Heart J.

[27] Pettersen E., Fredriksen P.M., Urheim S. (2009). Ventricular function in patients with transposition of the great arteries operated with arterial switch. Am J Cardiol.

[28] Engele L.J., van der Palen R.L.F., Egorova A.D. (2023). Cardiac fibrosis and innervation state in uncorrected and corrected transposition of the great arteries: a postmortem histological analysis and systematic review. J Cardiovasc Dev Dis.

[29] Hauser M., Bengel F.M., Kuhn A. (2001). Myocardial blood flow and flow reserve after coronary reimplantation in patients after arterial switch and ross operation. Circulation.

[30] Voges I., Jerosch-Herold M., Hedderich J. (2013). Implications of early aortic stiffening in patients with transposition of the great arteries after arterial switch operation. Circ Cardiovasc Imag..

[31] Meluzin J., Spinarova L., Hude P. (2005). Prognostic importance of various echocardiographic right ventricular functional parameters in patients with symptomatic heart failure. J Am Soc Echocardiogr.

[32] Grotenhuis H.B., Kroft L.J., van Elderen S.G. (2007). Right ventricular hypertrophy and diastolic dysfunction in arterial switch patients without pulmonary artery stenosis. Heart.

[33] Schuuring M.J., Bolmers P.P., Mulder B.J. (2012). Right ventricular function declines after cardiac surgery in adult patients with congenital heart disease. Int J Cardiovasc Imag.

[34] Koolbergen D.R., Manshanden J.S., Yazdanbakhsh A.P. (2014). Reoperation for neoaortic root pathology after the arterial switch operation. Eur J Cardio Thorac Surg.

[35] Giardini A., Khambadkone S., Rizzo N. (2009). Determinants of exercise capacity after arterial switch operation for transposition of the great arteries. Am J Cardiol.

[36] Luo D., Zheng X., Yang Z. (2023). Machine learning for clustering and postclosure outcome of adult CHD-PAH patients with borderline hemodynamics. J Heart Lung Transplant.

