# Supplementary material for: Using machine learning analysis to describe patterns in tissue Doppler and speckle tracking echocardiography in patients with transposition of the great arteries after arterial switch operation
Source: Int J Cardiol Congenit Heart Dis. 2024 Dec 20;19:100560. doi: 10.1016/j.ijcchd.2024.100560 (PMC11803126; doi:10.1016/j.ijcchd.2024.100560)
Supplement: Multimedia component 1 [file mmc1.docx]

**Table S1. Echocardiographic parameters in the study population, raw values.**

| **Variable** | **All patients (n=124)** | **TGA-IVS (n=94)** | **TGA-VSD (n=30)** | **P-value** |
| --- | --- | --- | --- | --- |
| **Conventional parameters** |  |  |  |  |
| LVEDd, mm | 43.6 ± 8.2 | 44.1 ± 8.0 | 45.6 ± 8.8 | 0.384 |
| LVEDs, mm | 27.5 ± 5.7 | 27.9 ± 5.4 | 29.1 ± 6.0 | 0.318 |
| FS, % | 36.5 ± 3.9 | 36.7 ± 3.86 | 36.0 ± 4.13 | 0.451 |
| LVEF, % | 51.6 ± 3.93 | 51.7 ± 3.6 | 51.1 ± 4.78 | 0.522 |
| TAPSE, mm | 16.1 ± 3.4 | 16.2 ± 3.4 | 15.9 ± 3.7 | 0.699 |
| MV E velocity, cm/s | 112 ± 18.2 | 113 ± 15.9 | 106 ± 22.3 | 0.093 |
| MV A velocity, cm/s | 48.9 ± 14.6 | 48.4 ± 14.7 | 48.5 ± 16.5 | 0.949 |
| MV E/A ratio | 2.5 ± 0.9 | 2.6 ± 0.9 | 2.39 ± 0.8 | 0.315 |
| TV E velocity, cm/s | 75.3 ± 15.3 | 74.8 ± 13.3 | 79.0 ± 19.4 | 0.225 |
| TV A velocity, cm/s | 46.0 ± 14.8 | 43.7 ± 13.2 | 50.5 ± 14.9 | **0.030** |
| TV E/A ratio | 1.8 ± 0.5 | 1.9 ± 0.5 | 1.67 ± 0.5 | 0.098 |
| **TDI parameters** |  |  |  |  |
| LV s' velocity, cm/s | 8.8 ± 2.0 | 8.7 ± 1.9 | 9.4 ± 2.2 | 0.174 |
| LV e' velocity, cm/s | 18.2 ± 3.0 | 18.3 ± 3.1 | 18.7 ± 2.6 | 0.506 |
| LV a' velocity, cm/s | 5.4 ± 1.6 | 5.2 ± 1.5 | 5.67 ± 1.6 | 0.144 |
| Septal s' velocity, cm/s | 6.4 ± 1.3 | 6.6 ± 1.2 | 6.4 ± 1.0 | 0.305 |
| Septal e' velocity, cm/s | 12.5 ± 2.3 | 13.1 ± 2.1 | 12.0 ± 2.2 | **0.019** |
| Septal a' velocity, cm/s | 5.5 ± 1.3 | 5.6 ± 1.2 | 5.3 ± 1.2 | 0.174 |
| RV s' velocity, cm/s | 8.8 ± 1.7 | 9.2 ± 1.6 | 8.5 ± 1.3 | **0.019** |
| RV e' velocity, cm/s | 12.8 ± 3.2 | 13.4 ± 3.1 | 11.9 ± 2.9 | **0.017** |
| RV a' velocity, cm/s | 5.9 ± 1.4 | 6.0 ± 1.5 | 5.8 ± 1.4 | 0.584 |
| LV E/e' ratio | 6.4 ± 1.5 | 6.4 ± 1.3 | 5.8 ± 1.4 | **0.044** |
| RV E/e' ratio | 6.2 ± 2.0 | 5.9 ± 1.7 | 6.8 ± 1.6 | **0.008** |
| **STE parameters of LV** |  |  |  |  |
| Basal septal LS, % | 16.0 ± 4.0 | 16.7 ± 3.7 | 15.6 ± 3.9 | 0.154 |
| Mid septal LS, % | 19.1 ± 3.2 | 19.8 ± 3.1 | 18.8 ± 2.6 | 0.079 |
| Apical septal LS, % | 16.1 ± 5.7 | 16.5 ± 5.3 | 15.0 ± 4.6 | 0.142 |
| Apical lateral LS, % | 16.5 ± 5.1 | 16.4 ± 4.7 | 16.6 ± 6.3 | 0.912 |
| Mid lateral LS, % | 15.2 ± 5.0 | 15.0 ± 4.8 | 15.7 ± 5.8 | 0.508 |
| Basal lateral LS, % | 18.0 ± 5.8 | 17.9 ± 5.7 | 17.8 ± 4.3 | 0.945 |
| Mean 4-chamber LS, % | 16.1 ± 2.8 | 16.3 ± 2.4 | 15.8 ± 3.0 | 0.332 |

*FS*, fractional shortening; *IVS*, intact ventricular septum; *LS*, longitudinal strain; *LV*, left ventricle/ventricular; *LVEDd*, left ventricular end-diastolic dimension; *LVEDs*, left ventricular end-systolic dimension; *LVEF*, left ventricular ejection fraction; *MV*, mitral valve; *RV*, right ventricle/ventricular; *STE*, speckle tracking echocardiography; *TAPSE*, tricuspid annular plane systolic excursion; *TDI*, tissue Doppler imaging; *TGA*, transposition of great arteries; *TV*, tricuspid valve.

Same remark on FS not in and EF ?
