# Supplementary material for: Using machine learning analysis to describe patterns in tissue Doppler and speckle tracking echocardiography in patients with transposition of the great arteries after arterial switch operation
Source: Int J Cardiol Congenit Heart Dis. 2024 Dec 20;19:100560. doi: 10.1016/j.ijcchd.2024.100560 (PMC11803126; doi:10.1016/j.ijcchd.2024.100560)
Supplement: Multimedia component 2 [file mmc2.docx]

**Table S2. Echocardiographic parameters of the clusters, raw values.**

| **Variable** | **Cluster 1 (n=37)** | **Cluster 2 (n=53)** | **Cluster 3 (n=34)** | **P-value overall** | **P-value 1 vs 2** | **P-value 1 vs 3** | **P-value 2 vs 3** |
| --- | --- | --- | --- | --- | --- | --- | --- |
| **Conventional parameters** |  |  |  |  |  |  |  |
| LVEDd, mm | 47.8 ± 7.62 | 38.5 ± 5.85 | 50.0 ± 5.82 | **<0.001** | **<0.001** | 0.328 | **<0.001** |
| LVEDs, mm | 29.7 ± 5.40 | 24.7 ± 4.14 | 31.9 ± 4.50 | **<0.001** | **<0.001** | 0.118 | **<0.001** |
| FS, % | 37.9 ± 3.92 | 35.8 ± 3.32 | 36.2 ± 4.47 | **0.035** | **0.032** | 0.141 | 0.907 |
| LVEF, % | 54.3 ± 3.72 | 50.3 ± 2.92 | 50.6 ± 4.15 | **<0.001** | **<0.001** | **<0.001** | 0.929 |
| TAPSE, mm | 17.8 ± 3.50 | 14.7 ± 3.05 | 16.6 ± 3.05 | **<0.001** | **<0.001** | 0.276 | **0.025** |
| MV E velocity, cm/s | 116 ± 19.2 | 110 ± 15.9 | 108 ± 18.7 | 0.141 | 0.301 | 0.138 | 0.807 |
| MV A velocity, cm/s | 50.1 ± 16.8 | 49.7 ± 16.2 | 45.0 ± 10.6 | 0.287 | 0.992 | 0.340 | 0.344 |
| MV E/A ratio | 2.61 ± 1.15 | 2.44 ± 0.78 | 2.51 ± 0.74 | 0.685 | 0.660 | 0.894 | 0.931 |
| TV E velocity, cm/s | 77.4 ± 14.8 | 74.4 ± 13.4 | 77.3 ± 17.7 | 0.551 | 0.613 | 0.999 | 0.651 |
| TV A velocity, cm/s | 44.1 ± 15.5 | 47.7 ± 12.7 | 43.2 ± 13.7 | 0.268 | 0.447 | 0.957 | 0.301 |
| TV E/A ratio | 1.94 ± 0.52 | 1.62 ± 0.39 | 1.89 ± 0.53 | **0.003** | **0.005** | 0.881 | **0.029** |
| **TDI parameters** |  |  |  |  |  |  |  |
| LV s' velocity, cm/s | 9.59 ± 2.15 | 8.25 ± 1.44 | 9.16 ± 2.26 | **0.004** | **0.004** | 0.604 | 0.080 |
| LV e' velocity, cm/s | 18.9 ± 3.02 | 17.9 ± 3.04 | 18.6 ± 2.70 | 0.246 | 0.248 | 0.910 | 0.506 |
| LV a' velocity, cm/s | 5.72 ± 1.52 | 4.99 ± 1.33 | 5.35 ± 1.65 | 0.076 | 0.062 | 0.544 | 0.524 |
| Septal s' velocity, cm/s | 7.58 ± 0.97 | 5.98 ± 0.82 | 6.24 ± 1.05 | **<0.001** | **<0.001** | **<0.001** | 0.430 |
| Septal e' velocity, cm/s | 13.8 ± 1.75 | 12.9 ± 2.03 | 11.7 ± 2.27 | **<0.001** | 0.105 | **<0.001** | **0.020** |
| Septal a' velocity, cm/s | 6.31 ± 1.23 | 5.18 ± 0.91 | 5.26 ± 1.26 | **<0.001** | **<0.001** | **<0.001** | 0.936 |
| RV s' velocity, cm/s | 14.1 ± 3.46 | 13.2 ± 2.66 | 11.6 ± 2.93 | **0.002** | 0.346 | **0.002** | **0.041** |
| RV e' velocity, cm/s | 6.59 ± 1.60 | 5.87 ± 1.25 | 5.37 ± 1.28 | **0.001** | **0.041** | **0.001** | 0.226 |
| RV a' velocity, cm/s | 10.4 ± 1.33 | 8.40 ± 1.10 | 8.52 ± 1.37 | **<0.001** | **<0.001** | **<0.001** | 0.886 |
| LV E/e' ratio | 6.29 ± 1.40 | 6.37 ± 1.51 | 5.93 ± 1.12 | 0.329 | 0.959 | 0.514 | 0.314 |
| RV E/e' ratio | 5.85 ± 1.80 | 5.86 ± 1.49 | 6.85 ± 1.76 | **0.013** | 1.000 | **0.033** | **0.020** |
| **STE parameters** |  |  |  |  |  |  |  |
| Basal septal LS, % | 17.4 ± 3.41 | 17.9 ± 3.04 | 13.1 ± 3.15 | **<0.001** | 0.746 | **<0.001** | **<0.001** |
| Mid septal LS, % | 20.7 ± 2.57 | 20.1 ± 2.46 | 17.4 ± 3.11 | **<0.001** | 0.598 | **<0.001** | **<0.001** |
| Apical septal LS, % | 19.3 ± 4.19 | 14.8 ± 4.67 | 15.0 ± 5.66 | **<0.001** | **<0.001** | **0.001** | 0.972 |
| Apical lateral LS, % | 17.7 ± 3.93 | 16.2 ± 5.15 | 15.5 ± 6.05 | 0.167 | 0.331 | 0.164 | 0.826 |
| Mid lateral LS, % | 16.3 ± 4.55 | 16.3 ± 5.12 | 12.2 ± 4.22 | **<0.001** | 0.999 | **0.001** | **<0.001** |
| Basal lateral LS, % | 18.2 ± 5.60 | 19.4 ± 5.19 | 15.4 ± 4.61 | **0.002** | 0.485 | 0.063 | **0.001** |
| Mean 4-chamber LS, % | 17.5 ± 2.07 | 16.7 ± 1.97 | 14.0 ± 2.44 | **<0.001** | 0.173 | **<0.001** | **<0.001** |

*FS*, fractional shortening; *LS*, longitudinal strain; *LV*, left ventricle/ventricular; *LVEDd*, left ventricular end-diastolic dimension; *LVEDs*, left ventricular end-systolic dimension; *LVEF*, left ventricular ejection fraction; *MV*, mitral valve; *RV*, right ventricle/ventricular; *STE*, speckle tracking echocardiography; *TAPSE*, tricuspid annular plane systolic excursion; *TDI*, tissue Doppler imaging; *TGA*, transposition of great arteries; *TV*, tricuspid valve.
